# Chromatin Molecular Complexes—Functional Organization, Protection and Regulation of the Genome

**DOI:** 10.3390/ijms23147516

**Published:** 2022-07-07

**Authors:** Ctirad Hofr

**Affiliations:** LifeB—Laboratory of Interaction and Function of Essential Biomolecules, Functional Genomics and Proteomics, National Centre for Biomolecular Research, Faculty of Science, Masaryk University, 611 37 Brno, Czech Republic; hofr@sci.muni.cz

This Special Issue highlights the advantages of using combined approaches to explore chromatin molecular complexes. The published findings provide insights into a range of molecular mechanisms which chromatin complexes employ in functionally interconnecting the world of nucleic acids and proteins. The main topic in this Special Issue is the activation of transcription. The authors investigated transcription activation at different levels—from straightforward nucleic acid structures to intricate cooperation pathways of high-order nucleoprotein complexes.

## 1. G-Quadruplexes as Epigenetic Regulators of Transcription

**Figure 1:**
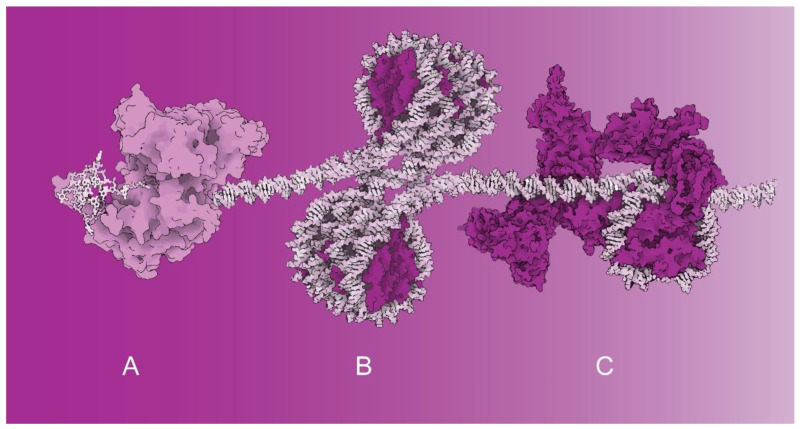
The molecular complexes regulating transcription activation that have been investigated within this Special Issue—illustrative examples based on known structures: (**A**) G-quadruplex processing via a helicase (PDB 5VHE). (**B**) Tetra-nucleosome activated for transcription (PDB 1ZBB). (**C**) Chromatin remodeler SWI/SNF bound to a nucleosome (PDB 6TDA). Structural representations of molecular complexes shown in (**A**–**C**) are not mutually proportional. Spatial models of nucleoprotein complexes were visualized using Protein Imager [3].

Special DNA arrangements into G-quadruplexes affect the initial phases of DNA transcription. Recent structural investigations of the Ferré-D’Amaré laboratory revealed the structural basis of G-quadruplex unfolding via helicases. The structural data suggest that the helicase rearranges its structure and pulls on the single-stranded DNA tail (Figure 1A). The helicase-induced structural rearrangements cause gradual G-quadruplex unfolding—one residue at a time [1]. The DNA sequences forming G-quadruplexes have been found at promoter regions and telomeres [2]. 

The Brazdova laboratory published immunofluorescence studies within this Special Issue, suggesting that G-quadruplexes are located in GC-rich euchromatin regions and outside the fibrillarin-positive compartments of nucleoli. When comparing several human and mouse cell lines, Komůrková et al. showed that G-quadruplexes occur most frequently in human embryonic stem cells which are more transcription-permissive than differentiated cells [4].

Moreover, the high degree of colocalization between G-quadruplexes and transcription factories suggests that G-quadruplexes and associated transcription factors cooperate during transcription initiation. In another independent study using ChIP-seq analysis with a G4 antibody, Lago et al. proposed that G-quadruplexes cooperate with associated transcription factors to determine cell-specific transcriptional programs [5]. Thus, G-quadruplexes are emerging as epigenetic regulators of transcription machinery.

## 2. Histone Variant H1.4 Phosphorylation—Role in Transcription Activation

Recently, the Cramer laboratory reported cryo-EM structural studies combined with electromobility shift assays showing that histone H1 binding to nucleosome arrays requires sufficiently long DNA linkers to enable H1 binding to arrayed nucleosomes. If DNA linkers are short, H1 cannot bind and structurally stabilize nucleosomes. Thus, transcription is enabled through the structural arrangement of the histones (Figure 1B) [6].

Another mechanism of transcription activation through histone phosphorylation has been suggested in this Special Issue. Saha et al. showed that the phosphorylated H1.4 histone variant remains associated with active promoters and may thus contribute to transcription activation [7]. Based on the collected genome-wide view of the H1.4 isoform phosphorylated in human breast cells derived from adenocarcinoma using specific antibodies, Saha et al. proposed that H1.4 phosphorylation at serine 187 via CDK9 is required to release promoter-proximal polymerases from beginning elongation by interacting directly with the polymerase or other parts of the transcription apparatus [7].

## 3. Chromatin Remodeling in Transcription Regulation

Two studies within the Special Issue have been dedicated to chromatin remodeling factors and their roles in transcription (Figure 1C). In the first study, Shidlovskii et al. [8] investigated an ATP-dependent chromatin remodeler from the switch/sucrose nonfermentable (SWI/SNF) family of proteins functioning mainly by opening the chromatin structure on promoters and enhancers. In vivo experiments employed the artificial tethering of two signature subunits, BAP170 and SAYP of the PBAP subfamily of chromatin remodelers. Functional studies in *Drosophila* strains have revealed that BAP170 plays an essential role in enhancer-dependent transcriptional activation mediated by PBAP chromatin remodelers [8].

The second publication regarding chromatin remodeling factors focused on the interaction between the SWI/SNF chromatin remodeling complex and transcription factor CTCF. Valletta et al. [9] used an affinity pull-down approach combined with liquid chromatography with tandem mass spectrometry to map the association of subunits of SWI/SNF chromatin remodeling complex with CTCF—a zinc finger CCCTC-binding factor.

The high occurrence of peptides from BRG1—the major ATPase subunit of the SWI/SNF complex during CTCF immunoprecipitation—has been detected. After the subsequent validation of selected subunits of the SWI/SNF complex via Western blot and ChIP-seq analyses, quantitative binding studies showed that the BRK domain of BRG1 directly interacts with CTCF at the region of zinc finger motifs 4–8 [9]. The published study employing complementary methodologies elucidated the interplay between the SWI/SNF remodeling complex and CTCF as architectural factors regulating DNA folding and gene expression.

Previous proteomic and bioinformatic studies revealed mutations of the SWI/SNF complex in over 20% of human cancers [10]. The importance of detailed studies of chromatin remodeling mechanisms was documented in recently developed SWI/SNF ATPase inhibitors acting as chromatin-targeted therapeutics for specific therapies of enhancer-addicted cancers [11].

## 4. Chromatin Molecular Complexes Reviewed—Structure and Compaction Perspective

Within this Special Issue, Morrison and Thakur reviewed distinct chromatin factors and molecular complexes that constitute (i) euchromatin as an open chromatin structure associated with active transcription, (ii) heterochromatin as a less accessible chromatin associated with silencing and (iii) centromeric chromatin as the site of spindle binding in chromosome segregation [12]. 

In the review section devoted to euchromatin, it was described that even though euchromatin forms only 8% of chromatin, euchromatin regulates the transcription of genes differentially and tightly via chromatin remodelers, histone modifications, histone variants and chaperones [12,13].

In the concluding remarks dedicated to heterochromatin, Morrison and Thakur asked the appealing question of what the recent research focus on the phase separation properties of heterochromatin adds to our knowledge of the molecular machinery of compaction, spatial arrangements and the function of heterochromatin.

Finally, in the centromeric chromatin section, it was described how highly specific chromatin-associated proteins participate in performing chromosome segregation. Centromeric chromatin is presented as a chromatin containing one of the least-conserved histone variants.

In summary, the articles published within this Special Issue contribute to our understanding of the fundamental mechanisms underlying the regulation of gene transcription and chromatin remodeling which could be employed in the localization, regulation and prevention of transcription, especially for diseases with peculiar transcription patterns.
